# Prior Image Guided Undersampled Dual Energy Reconstruction with Piecewise Polynomial Function Constraint

**DOI:** 10.1155/2013/437917

**Published:** 2013-10-27

**Authors:** Dufan Wu, Li Zhang, Liang Li, Le Shen, Yuxiang Xing

**Affiliations:** ^1^Key Laboratory of Particle & Radiation Imaging, Tsinghua University, Ministry of Education, Beijing 100084, China; ^2^Department of Engineering Physics, Tsinghua University, Beijing 100084, China

## Abstract

Dual energy CT has the ability to give more information about the test object by reconstructing the attenuation factors under different energies. These images under different energies share identical structures but different attenuation factors. By referring to the fully sampled low-energy image, we show that it is possible to greatly reduce the sampling rate of the high-energy image in order to lower dose. To compensate the attenuation factor difference between the two modalities, we use piecewise polynomial fitting to fit the low-energy image to the high-energy image. During the reconstruction, the result is constrained by its distance to the fitted image, and the structural information thus can be preserved. An ASD-POCS-based optimization schedule is proposed to solve the problem, and numerical simulations are taken to verify the algorithm.

## 1. Introduction

Computed tomography has become an important nondestructive detection method in medicine, industry, and security. Typically CT scans the object by a single energy to reconstruct the attenuation factors in order to evaluate the density distributions inside the test object. However, some materials' attenuation factors are close and hard to distinguish, which brings trouble for diagnosis. Since the attenuation factors are different under different X-ray energies, DECT [1] has been brought about to enhance the material distinguishing ability in CT. Furthermore, atomic numbers, electron densities, or specific material equivalent densities can also be reconstructed from DECT for better visualization. 

In DECT, the test object is scanned under different energies while keeping the object fixed. Thus, two different images, the low-energy image **x**_*L*_ and the high-energy image **x**_*H*_ can be reconstructed independently from the two sets of projections, the low-energy projections **p**_*L*_ and the high-energy projections **p**_*H*_. Although there are various techniques for DECT reconstruction, for example, prereconstruction [2], postreconstruction [3], and iterative reconstruction [4], we concentrate on reconstructing **x**_*L*_ and **x**_*H*_ in this paper to demonstrate the mathematics of our method. 

Dose has been concerned more and more recently with the increasing public awareness of the possible risks brought about by the radiation of CT scans. One of the most efficient ways to reduce dose is to reduce the sampling number. According to the concept of compressed sensing (CS) [5, 6], when the sampling numbers are reduced beneath the conventional required sampling rate, one can still accurately recover the signal by incorporating prior knowledge to the reconstruction. In the instance of DECT, **x**_*L*_ and **x**_*H*_ share identical structures because they are taken from the same object at almost the same time. One of the straightforward strategies for dose reduction is to undersample **x**_*H*_ while keeping **x**_*L*_ fully sampled. During the reconstruction of **x**_*H*_, the structural information extracted from the well-reconstructed **x**_*L*_ can be made utility to improve the reconstruction quality. 

Although the two images share the same structure, their attenuation factors are different under the two different energies, which leads to the grey scale difference in the reconstructed images. Similar situations can be found in multimodality imaging, where the grey scale values of the images are far different from each other. Bowsher prior has been used for MR/SPECT imaging to improve the reconstruction quality of SPECT [7, 8]. For DECT, Liu et al. used image segmentation on the fully sampled **x**_*L*_ to reduce the number of variables during the reconstruction of **x**_*H*_ [9]. Recently, some methods invoking CS have been proposed for DECT. Szczykutowicz and Chen applied PICCS to a slow kVp switching acquisition scheme and achieved good results [10, 11]. Xing and Zheng applied ART-TV on the ratio image of **x**_*L*_ to **x**_*H*_ for sparser presentation of the image [12].

It has been shown that invoking reference images in CS-based reconstruction is able to improve the reconstruction quality, but grey scale value compensation remains a challenge in DECT. For example, PICCS algorithm requires the reference image and the target image to be as close as possible, but the attenuation factor may differ widely under different energies. The ratio image method, on the other hand, requires that the changes in the grey scale values are proportional so that there are less edges in the ratio image and its gradient image is sparser. However, the change of the attenuation factor under different energies is unpredictable and the conditions required for the above methods may be violated sometimes. 

Here, we propose a novel CS-based method for undersampled DECT reconstruction. The well-reconstructed low-energy image **x**_*L*_ is used to sparsify the undersampled high-energy image **x**_*H*_. To compensate for the grey value scale difference, both images are divided into patches and polynomial fitting is used on each pair of patches to fit the reference image **x**_*L*_ to the target image **x**_*H*_. An l1-norm constraint is applied on the distance from **x**_*H*_ to the fitted image, and ASD-POCS [13] is adopted for the optimization. Since the piecewise polynomial fitting is able to almost precisely approximate the target image under most occasions, promising results can be achieved when the sampling rate is greatly reduced. 

The method is very much motivated by one of our previous works [14], feature constrained compressed sensing (FCCS). In the FCCS method, linear space extracted from training image set is used as the prior knowledge rather than a single image. During the reconstruction, the distance between the target image and the prior space is used as the constraint for the result. In this paper, the prior space is generated from a single reference image by taking its powers. The constraint on the distance between the target image and the polynomial space is achieved by piecewise polynomial fitting. 

The paper is organized as follows. In Section 2, the mathematical principles of the algorithm are presented. Numerical simulation results are shown in Section 3.  Section 4 is conclusions and discussions. 

## 2. Methodology

### 2.1. An Overview of the Method

The reconstruction formula of piecewise polynomial function constrained (PPFC) method is as follows:

(1) min⁡ ||xH−φ(xL,xH)||1s.t. ||AxH−pH||2≤ε         xH≥0,

where **φ**(**x**_*L*_, **x**_*H*_) is the piecewise polynomial fitting (PPF) which approximates **x**_*H*_ by **x**_*L*_. The images are represented by the row vectors **x**_*H*_ and **x**_*L*_. **A** is the system matrix, and **p**_*H*_ is the high-energy projections. After a high-quality **x**_*L*_ is reconstructed from the fully sampled low-energy projections, it is used as the prior knowledge to iteratively solve **x**_*H*_ form the undersampled **p**_*H*_ according to (1) by ASD-POCS algorithm. 

### 2.2. Image Approximation by Polynomial Fitting

Before introducing PPF, we will firstly show the least squares polynomial fitting method and some of its properties. In DECT, one of the ways to sparsify **x**_*H*_ by **x**_*L*_ is to find a transform **φ**(·) which makes ||**x**_*H*_−**φ**(**x**_*L*_,**x**_*H*_)||_0_ small. We will show that piecewise polynomial fitting is a good way to approximate **x**_*H*_ by **x**_*L*_, which actually makes the difference between them sparse. 

Image approximation by polynomial fitting is to solve the following equation:

(2) $$\begin{array}{c} \min{||x_{H}-f\left(x_{L},x_{H}\right)||}_{2}, \end{array}$$

where

(3) $$\begin{array}{c} f\left(x_{L},x_{H}\right)=\overset{p-1}{\underset{k=0}{\sum }}a_{k}x_{L}^{k}, \end{array}$$

where **x** ^*k*^ is defined as the element by element power of **x** and **x** ^0^ is an all-ones vector. (*p* − 1) is the order of the polynomial and *a*_0_ to *a*_*p*−1_ are the corresponding coefficients which are determined by both **x**_*H*_ and **x**_*L*_.

Equation (2) can be written into matrix form, which is

(4) $$\begin{array}{c} a=\underset{a}{\arg\;\min}{||V^{T}a-x_{H}||}_{2}, \end{array}$$

where

(5) $$\begin{array}{c} V=\left(\begin{array}{cccc} 1 & 1 & \cdots & 1\\ {\left(x_{L}\right)}_{1} & {\left(x_{L}\right)}_{2} & \cdots & {\left(x_{L}\right)}_{n}\\ \vdots & \vdots & \ddots & \vdots \\ {\left(x_{L}\right)}_{1}^{p-1} & {\left(x_{L}\right)}_{2}^{p-1} & \cdots & {\left(x_{L}\right)}_{n}^{p-1} \end{array}\right) \end{array}$$

is an order *p* Vandermonde matrix. (**x**_*L*_)_*i*_ means the *i*th element of **x**_*L*_. To further reduce the scale of (4), we adopted the following method for least squares solution:

(6) $$\begin{array}{c} Ha=Vx_{H}, \end{array}$$

where

(7) $$\begin{array}{c} H_{ij}:={\left(VV^{T}\right)}_{ij}=\overset{n}{\underset{k=1}{\sum }}{\left(x_{L}\right)}_{k}^{i+j-2} \end{array}$$

is an order *p* Hankel matrix. Although (6) is not the most numerically stable solution for least squares problems, it is enough for low-order polynomial fitting problems. Efficient algorithms such as the Gohberg-Kailath-Koltracht (GKK) algorithm [15] can be used to solve (6). The approximation result can be expressed as

(8) $$\begin{array}{c} f\left(x_{L},x_{H}\right)=V^{T}a=V^{T}H^{-1}Vx_{H}=P\left(x_{L}\right)x_{H}. \end{array}$$

The approximation has some good properties. Firstly, low-order polynomials have the property of smoothness, which maps similar values in **x**_*L*_ to similar values in **f**(**x**_*L*_, **x**_*H*_). Thus, the fitting result will not be seriously influenced by noises. Secondly, if there are not too many kinds of values presented in the images, low-order polynomials can always guarantee a good approximation. For example, if there are only 5 pairs of values presented in **x**_*L*_ and **x**_*H*_, an polynomial of order 4 is able to precisely map **x**_*L*_ to **x**_*H*_. If the values distribute around a few points, the approximation error will also remain small. Furthermore, low-order polynomial fitting is numerical stable. High-order least squares polynomial fitting requires high computational precision, and the result will be sensitive to rounding error. To solve this problem, we divide the image into small patches so that there are not too many kinds of grey values in each patch, and low-order polynomial fitting is applicable. Last but not least, the least squares polynomial fitting problem can be efficiently solved by the GKK algorithm, whose time and space complexities are *O*(*np*) + *O*(*p* ^2^) and *O*(*n*) + *O*(*p* ^2^). Since the polynomial order *p* is small, the method is both time and memory saving.

In our experiments, the order *p* is set to 4, which is the minimum value required for fitting in our case. A small order makes computation with single floating point data type feasible. A higher order is applicable, but it means heavier computational load. It may be preferred when the test object is more complex; for example, the object contains more kinds of materials in a small region.

### 2.3. Piecewise Polynomial Fitting for the Entire Image

As we have stated, low-order polynomial fitting requires that the grey values of the image distribute around only a few points. Thus, the size of the image for fitting should be small for accuracy. This can be achieved by extracting patches from the original image and applying polynomial fitting on the sub images. On the other hand, the patches should be big enough to hold the structural information with them. There should also be adequate overlapping areas between adjacent patches or the difference between patches will lead to obvious block-like artifacts in the approximated image. In our experiments, the patch size is selected as 16 by 16, and the offset of adjacent patches is 4 by 4, which leads to an overlapping size of 12 by 12.

**Figure 1:**
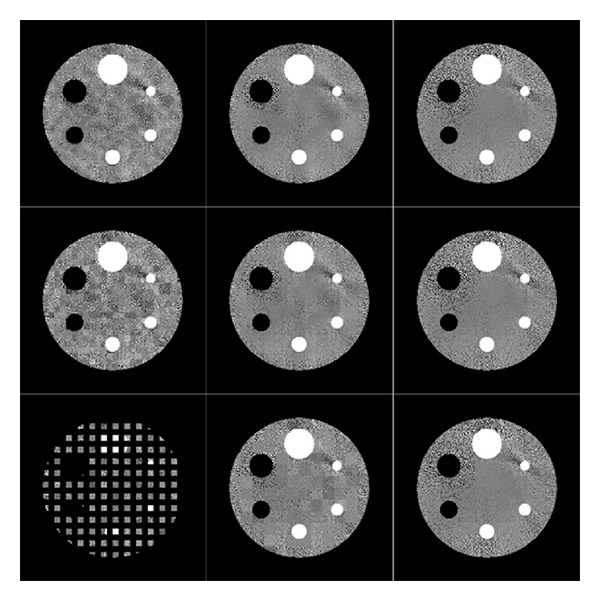
The PPF results on noisy phantom with different patch sizes and offsets. From the top row to the bottom row, the corresponding offsets between adjacent patches are 4, 8, and 16. From the leftmost column to the rightmost column, the corresponding patch sizes are 8, 16, and 32. The grey scale value window is [0.185, 0.192].

Figure 1 shows the PPF results on noisy phantom with different patch sizes and offsets. Noisy **x**_*L*_ is used to fit the noisy **x**_*H*_. Obvious block-like artifacts can be observed when the patch size and offset are (8,8) and (16, 16). This is because in each small patch, the noise distributions are not identical, and without proper smoothing between adjacent patches, block-like artifacts emerge. Furthermore, the patch size should be larger than the offset; otherwise, part of the image will be missing in the result (see the result at the bottom left corner of Figure 1).

When approximating **x**_*H*_ by **x**_*L*_ with PPF, for each pair of patches, the high-energy image patch **x**_*H*_^(*s*)^ is approximated by the low-energy image patch **x**_*L*_^(*s*)^ according to (8). Then the entire image is generated by aggregating the patches. Define the patch selection matrix **E** ^(*s*)^ which satisfies

(9) $$\begin{array}{c} x_{H}^{\left(s\right)}=E^{\left(s\right)}x_{H}. \end{array}$$

Then putting the patch back to its original position can be achieved by the transposed matrix **E** ^(*s*)*T*^. The aggregation can be expressed as

(10) φ(xL,xH)=∑sE(s)TGP(xL(s))E(s)xH∑sE(s)TGE(s)·1=(∑sE(s)TW(s)P(xL(s))E(s))xH=BxH.

In (10), the division is element by element. **G** is a Gaussian kernel which is used for smoothness during aggregation. **W** ^(*s*)^ is the normalized weighting for each patch. It is a diagonal matrix and

(11) $$\begin{array}{c} W_{ii}^{\left(s\right)}=\frac{1}{{\left(E^{\left(s\right)}\sum_{l}^{}E^{(l)T}GE^{\left(l\right)}\cdot 1\right)}_{i}}G_{ii}, \end{array}$$

which can be calculated by dividing the Gaussian kernel **G** by the aggregated weightings. 

The transpose of **B** is

(12) $$\begin{array}{c} B^{T}=\underset{s}{\sum }E^{(s)T}P^{T}\left(x_{L}^{\left(s\right)}\right)W^{(s)T}E^{\left(s\right)}. \end{array}$$

Since **P** = **V** ^*T*^ **H** ^−1^ **V** = **V** ^*T*^(**V** **V** ^*T*^)^−1^ **V** is a symmetric matrix, **P** ^*T*^ = **P** holds. Furthermore, **W** ^(*s*)^ is diagonal, so it also holds that **W** ^(*s*)*T*^ = **W** ^(*s*)^. The transposed matrix can be written as

(13) $$\begin{array}{c} B^{T}=\underset{s}{\sum }E^{(s)T}P\left(x_{L}^{\left(s\right)}\right)W^{\left(s\right)}E^{\left(s\right)}. \end{array}$$

The transposed operator will be of future use and we will take a look at its properties here. The PPF matrix **B** is realized by first fitting the patches and then weighted aggregation, but the transposed PPF matrix **B** ^*T*^ is realized by first weighting the patches, then fitting, and then unweighted aggregation. The algorithms for PPF and its transpose are shown in Algorithms 1 and 2.

*δ* is a small value in case the values in **x**_*L*_^(*s*)^ are almost zeros and the polynomial fitting algorithm fails. It is set at 1 × 10^−4^ in our experiment.

### 2.4. Optimization by ASD-POCS

The formula for optimization is shown in (1). When adapting ASD-POCS algorithm to the problem, the only change to make is the first derivative of the objective function. The objective function is

(14) $$\begin{array}{c} g\left(x_{H}\right)={||x_{H}-\varphi \left(x_{L},x_{H}\right)||}_{1}={||\left(I-B\right)x_{H}||}_{1}. \end{array}$$

Using the chain derivative rule, one can easily derive the gradient of the objective function:

(15) $$\begin{array}{c} \nabla g\left(x_{H}\right)=\left(I-B^{T}\right)\mathrm{sgn}\left(\left(I-B\right)x_{H}\right), \end{array}$$

where sgn⁡(·) is the element by element sign function:

(16) $$\begin{array}{c} {\mathrm{sgn}}_{i}\left(x\right)=\{\begin{array}{cc} -1 & x_{i}\leq -\delta_{2},\\ \frac{x_{i}}{\delta_{2}} & -\delta_{2}<x_{i}\leq \delta_{2},\\ 1 & x_{i}>\delta_{2}, \end{array} \end{array}$$

where *δ*_2_ is a small regularization factor near zero. In our experiments, this value is set at 0.01.

The initial value for the iterations is crucial for the convergence speed. For PPFC, 10 times SART is used to estimate **x**_*H*_ and PPF is used to approximate the current image ${\hat{x}}_{H}$ by **x**_*L*_. The approximation result $\varphi (x_{L},{\hat{x}}_{H})$ is used as the initial value of the iterations. 

**Figure 2:**
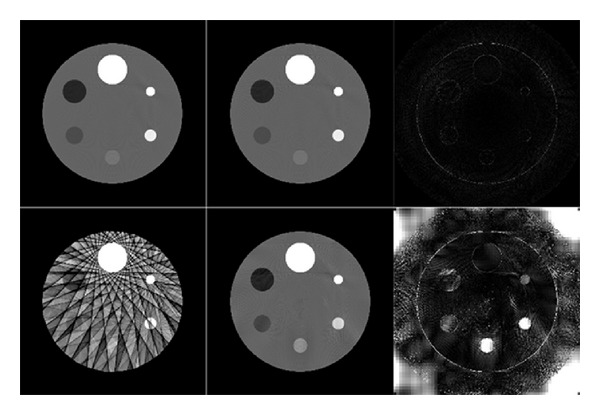
The approximation result of PPF. **x**_*L*_ is used to approximate **x**_*H*_. The top row is fully sampled. The bottom row is sparsely sampled by 15 projections. The left most column is the reconstruction result by FBP. The middle column is the PPF approximation results. The right most column is the difference between the reconstructed images and the approximation results, which is the PPFC transform. The grey scale window for the first and second columns is [0.15, 0.25]. For the third column, the window is [0, 0.01].

The sparsity of the PPFC transform (**I** − **B**)**x**_*H*_ is shown in Figure 2. It can be seen that the PPFC transform on the well-reconstructed image is sparse, while the zero entities are much more in the image with artifacts.

## 3. Simulations

### 3.1. Simulation Method

**Figure 3:**
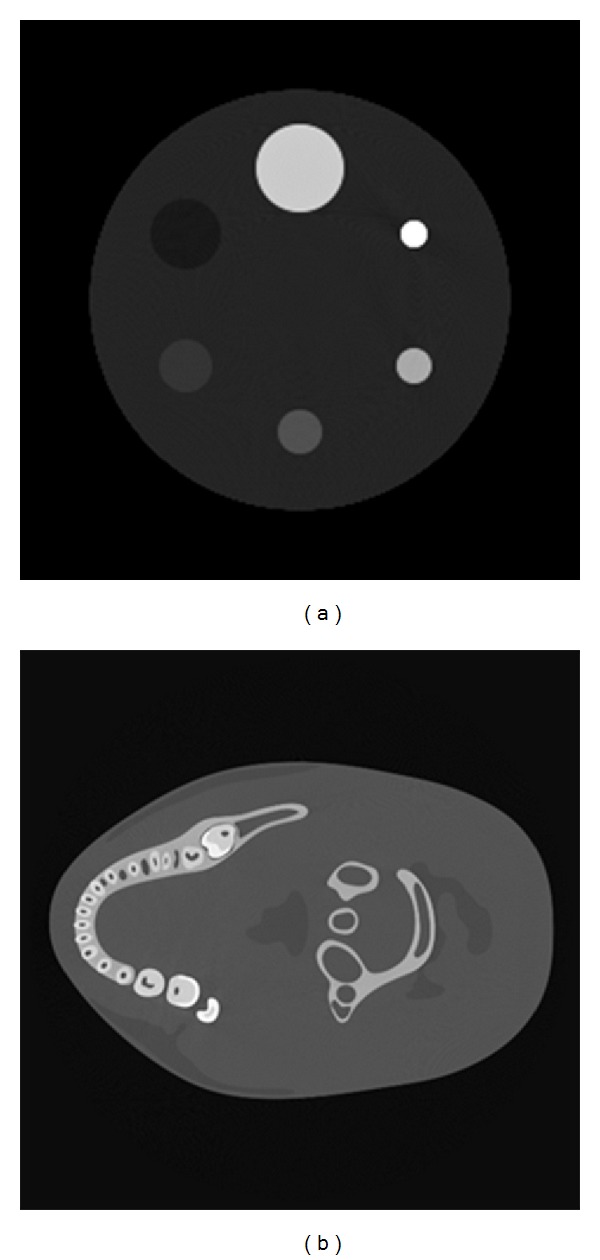
The phantoms used for simulation: (a) the cylinder phantom; the base cylinder is PMMA and the inner cylinders from the biggest to the smallest are made of bone, water, and 1%, 2%, 5%, and 10% NaI solutions and (b) the dental phantom.

Multienergy projections are used for the experiments. The spectrum of the X-ray source is generated by the Monte Carlo method. Three different spectrums are used for the experiments: a 90 kVp spectrum, a 120 kVp spectrum, and a beam-hardened 160 kVp spectrum. There are two different phantoms for testing, a cylinder phantom and a realistic dental phantom. The cylinder phantom is forward projected by the 90 kVp spectrum and the 160 kVp spectrum. The energy used for the dental phantom is 90 kVp and 120 kVp. The low-energy image **x**_*L*_ is reconstructed by FBP and the high-energy image **x**_*H*_ is reconstructed by the proposed PPFC method with only 15 projections being uniformly sampled on 360 degrees. PICCS is also realized under the ASD-POCS framework as a reference method and its initial value is set at the energy normalized image $\left({||{\hat{x}}_{H}||}_{2}/{||x_{L}||}_{2}\right)x_{L}$. The simulation phantoms are shown in Figure 3.

### 3.2. Cylinder Phantom Experiments

**Figure 4:**
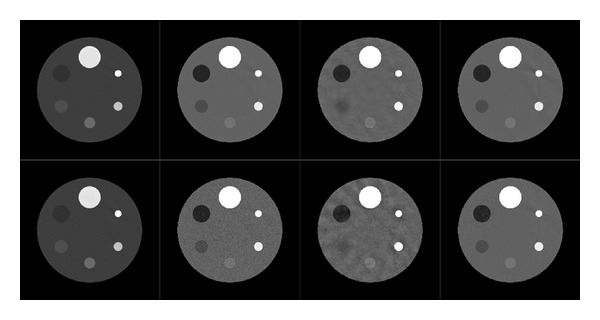
The reconstruction results of cylinder phantoms. The top row is the results from noiseless projections and the bottom row is the results from noisy projections. From left to right: the first column is the fully sampled **x**_*L*_ reconstructed by FBP, the second column is the fully sampled **x**_*H*_ reconstructed by FBP, the third column is the reconstruction results of undersampled **x**_*H*_ by PICCS, and the fourth column is the results from PPFC, the grey scale windows for the low-energy images and high-energy images are [0.1, 0.6] and [0.15, 0.25].

The cylinder phantom is first forward projected by the 90 kVp and 160 kVp spectrums with fan beam geometry to get the high and low projections **p**_*H*_ and **p**_*L*_. Then the low-energy image **x**_*L*_ is reconstructed from 900 projections by FBP. The region outside the ROI of FBP is set at zero. For the high-energy image **x**_*H*_, only 15 projections uniformly sampled across 360 degrees are used for reconstruction. Then both PPFC and PICCS are employed to reconstruct the phantom. Furthermore, the algorithms are also tested against noises. The noises are added to the projections by setting the photon number of the X-ray source at 2 × 10^5^ per detector bin per projection. The scan geometry is shown in Table 1, and the simulation results are shown in Figure 4 and Table 2.

In the results of PICCS, the cylinder made of 1% NaI solution is blurred. The reason is that in the reference image **x**_*L*_, the attenuation factor of the 1% NaI solution is larger than the background PMMA cylinder, but in the target image **x**_*H*_, the attenuation factor of the 1% NaI solution becomes smaller than the PMMA's attenuation factor. The way PICCS uses the prior image by subtraction actually reduces the sparsity on the cylinder of 1% NaI solution. As its consequence, PICCS is not able to recover the cylinder of 1% NaI solution well while other cylinders are well preserved. 

As for the results of PPFC, all the cylinders including the 1% NaI solution cylinder are well recovered. Furthermore, the results of the noisy projections have not degraded much comparing to the noiseless results. Thus, the experiments indicate that PPFC is compensating grey scale values difference and noise stable. 

### 3.3. Realistic Phantom Experiments

**Figure 5:**
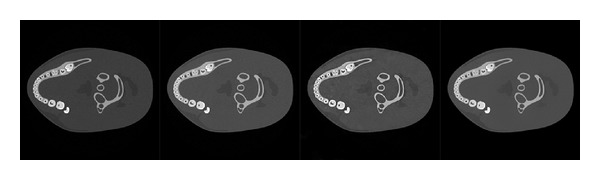
The reconstruction results of the dental phantom. From left to right: the first one is the **x**_*L*_ reconstructed from 720 projections by FBP, the second one is the **x**_*H*_ reconstructed from 70 projections by FBP, the third one is the results by PICCS from 15 projections, and the last one is the results of PPFC. The grey scale windows for the low and high-energy images are [0, 0.85] and [0, 0.65]. All the results are reconstructed from the noisy projections.

The dental phantom is forward projected by the 90 kVp and 120 kVp spectrums with fan beam geometry. The low-energy image **x**_*L*_ is reconstructed by FBP from 720 projections. **x**_*H*_ is downsampled to 15 projections and reconstructed by PPFC and PICCS. Noises with 1 × 10^5^ initial photons are also added to the projections. The scan geometry is shown in Table 3, and the corresponding results are shown in Figure 5 and Table 4. 

The experiments on the realistic phantom show that PPFC is able to reconstruct objects with complicated structures as well as the simple objects. It also shows an advantage over PICCS on the aspect of RMSE. 

## 4. Conclusion and Discussions

In this paper, we propose a CS-based method for undersampled DECT reconstruction with piecewise polynomial function constraint. The low-energy image is reconstructed from fully sampled projections and the high-energy image is reconstructed from severely corrupted samples with the well-reconstructed low-energy image as the reference. The proposed piecewise polynomial fitting method has good ability to compensate for the grey scale value difference between the high- and low-energy images. Under most conditions, the target image can be well approximated by the reference image using the PPF, which ensures the sparsity of the PPFC transform. The simulation results show that our method is both accurate and stable. 

The drawback of the algorithm is that the piecewise polynomial fitting is still not efficient enough. However, the fitting of each patch is independent and the algorithm can be further accelerated by parallel computation. Furthermore, the algorithm has the potential to reconstruct the decomposition coefficients images in DECT, whose values are far from the values in the low-energy image. Applying the method to a dual-effect or dual-material decomposition reconstruction is of future concerns.

## Figures and Tables

**Algorithm 1:**
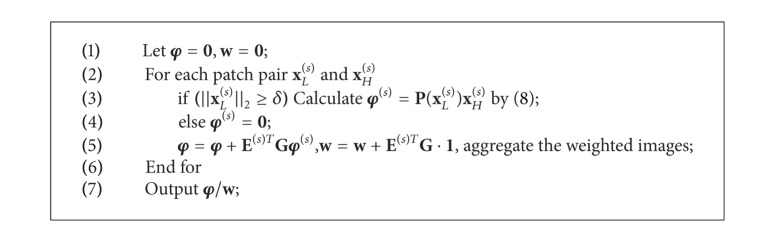
Image approximation by PPF.

**Algorithm 2:**
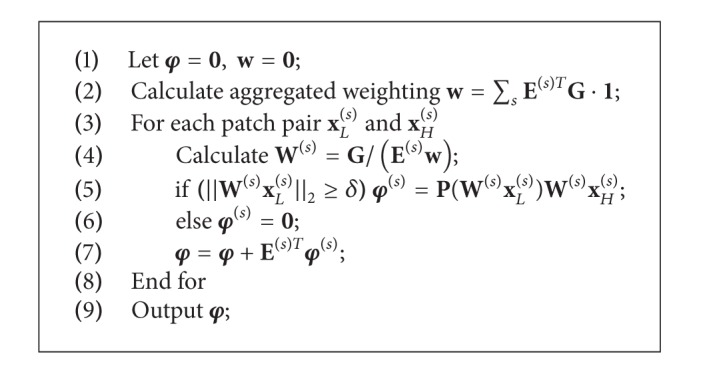
Transposed PPF.

**Table 1:** The scan geometry for the cylinder phantom.

Parameter	Value
Source to center distance	75 cm
Source to detector distance	105 cm
Total width of detector	9.6 cm
Number of detector bins	512
Reconstruction resolution	256 × 256
Reconstruction size	6.4 cm × 6.4 cm

**Table 2:** The quantitative estimations of the reconstruction results of the cylinder phantom.

Method	RMSE	Iteration number	Time (s)
Noiseless PPFC	2.9 × 10^−4^	10	31.2
Noiseless PICCS	4.8 × 10^−4^	100	34.3
Noisy PPFC	2.9 × 10^−4^	10	31.2
Noisy PICCS	8.7 × 10^−4^	100	34.4

The RMSE is calculated by comparing to the noiseless result of FBP.

The iterations numbers do not include the 10 times pre-iterations and are enough for convergence.

The code is implemented by C++ on a machine with Intel Core i5 processor and 3 GB memory.

**Table 3:** The scan geometry for the dental phantom.

Parameter	Value
Source to center distance	50 cm
Source to detector distance	80 cm
Total width of detector	30 cm
Number of detector bins	960
Reconstruction resolution	512 × 512
Reconstruction size	19.2 cm × 19.2 cm

**Table 4:** The quantitative estimations of the reconstruction results of the dental phantom.

Method	RMSE	Iteration number	Time (s)
PPFC	1.1 × 10^−3^	20	238
PICCS	2.5 × 10^−3^	200	288

The RMSE is compared to the noiseless results of FBP.
